# Incidence of Leptospirosis infection in the East Zone of Sao Paulo City, Brazil

**DOI:** 10.1186/1755-7682-6-23

**Published:** 2013-05-14

**Authors:** Kátia Eiko Miyazato, Alexandre LA Fonseca, Luciana Z Caputto, Katya C Rocha, Ligia A Azzalis, Virginia BC Junqueira, Edimar C Pereira, Loide C Chaves, David Feder, Roseli Corazzini, Luiz Carlos De Abreu, Vitor E Valenti, Sheylla Nadjane Batista Lacerda, Flávia C Goulart, Fernando LA Fonseca

**Affiliations:** 1 IPESSP (Instituto Paulista de Ensino em Saúde de São Paulo), Alameda Franca, 1604, São Paulo, SP, 01422-001, Brazil; 2 Departamento de Patologia, Faculdade de Medicina do ABC, Av. Príncipe de Gales, 821, Santo André, SP, 09060-650, Brazil; 3 Instituto de Ciências Químicas, Ambientais e Farmacêuticas, Universidade Federal de São Paulo, UNIFESP, Rua Prof. Artur Riedel, 275, Diadema 09972-270, Brazil; 4 Departamento de Enfermagem, Faculdade de Medicina do ABC, Av. Príncipe de Gales, 821, Santo André, SP, 09060-650, Brazil; 5 Departamento de Morfologia e Fisiologia, Faculdade de Medicina do ABC, Av. Príncipe de Gales, 821, Santo André, SP 09060-650, Brazil; 6 Departamento de Fonoaudiologia, Faculdade de Filosofia e Ciências, Universidade Estadual Paulista, UNESP, Av. Hygino Muzzi Filho, 737, Marília, SP, 17.525-900, Brazil

**Keywords:** Epidemiological survey, Precipitation index, Points of flooding, Laboratory methods

## Abstract

**Background:**

Leptospirosis is a zoonosis which is spread through contamined running water. This contaminations is seriously affected by the flooding which occurs in the area surrounding the Aricanduva river. The transmission of the disease results mainly from the contact of water with soil contaminated by the urine of infected animals. We aimed to conduct an epidemiological survey on Leptospirosis cases in Sao Paulo East Zone area.

**Method:**

The analysis conducted in this study was based on data collected from the health authorities of that region close the Aricanduva river between 2007 and 2008 years, which give the rates of confirmed cases, mortality and death from human Leptospirosis. Other information concerned with the relationships among rainfall index, points of flooding and incidence of Leptospirosis.

**Results:**

We observed a direct and important water contamination. Records of flooding points and dates of the reported cases in the region showed a direct relationship from which the period of higher rainfall also recorded an increase in cases. The annual record of the city and the region and rainfall regions also presented correlation.

**Conclusion:**

The association between the indices of flooding and Leptospirosis cases indicates that preventive measures are necessary to avoid exposing the community.

## Background

Leptospirosis is a zoonosis (disease transmitted by animals), acute and infectious disease caused by the bacterium Leptospira interrogans [1]. According to the manual of the Brazilian Secretariat of Health Surveillance [2], Leptospirosis is transmitted to humans by the urine of mice and rats. After heavy rains, the water carries bacteria from rats’ and mice’s urines to homes and public roads. Generally, outbreaks of Leptospirosis begin one week after the floods [3].

The number of human cases ranges in the year based on higher rainfall and according to the sanitation infrastructure of the region. In urban areas, the most contact with the agent occurs mainly during flooding periods [2].

Different areas in Brazil are affected by Leptospirosis. For instance, the state of Rio Grande do Sul has a high incidence of leptospirosis, with about 10 cases per 100 000 inhabitants, higher than the national average (3.5 cases per 100 000 inhabitants). Most cases (86%) correspond to males and rural residents (69%). The probable location of infection, indicated by epidemiological investigation of cases indicates both the workplace and home as a principal means of contact with the agent [2].

In the East zone of São Paulo, the area of the Aricanduva river belongs entirely to Sao Paulo city, comprising all or part of the Districts of Penha, Birmingham, Carrao, Vila Matilde, Aricanduva, Vila Formosa, City Leader, Park Carmo, Jose Bonifacio, Sapopemba, Sao Matheus, Iguatemi and Sao Rafael. Regionally, these districts are administered by the AR’s (Regional Health Administration) of Aricanduva/Vila Formosa, Penha, Bristol, Itaquera, Uxbridge and Sao Matheus [4].

According to data obtained from the São Paulo city hall [5], the Aricanduva river is responsible for draining 100km^2^, covering the districts of Tatuape, Vila Formosa, Carrao, Sao Matheus, Vila Matilde, Penha, Cidade líder, Itaquera and Parque do Carmo. These neighborhoods are part of the subdistricts of Aricanduva (262,255 inhabitants) Sao Matheus (409,478 inhabitants), Penha (475,678 inhabitants) and Itaquera (502,823 inhabitants), totaling 1,643,134 inhabitants [6].

The region of the Aricanduva river has one of the greatest population densities of Sao Paulo city and over the past two decades it lost more than 50% of permeable areas. As a consequence, it increased the volume of water in the Aricanduva river over its capacity, which provoked episodes of flooding [5].

Although this area is often committed by strong rain, the literature did not report yet if there is any association between rain intensity and Leptospirosis incidence around Aricanduva river. In addition, previous studies on general infection have been received great attention worldwide [7-10]. We believe that the exposure of findings regarding this issue would help to elaborate new procedures and laws to improve the conditions in which the population currently lives. Therefore, we aimed to evaluate epidemiological data of Leptospirosis cases in the region of Sao Paulo East zone, SP, Brazil and to investigate the association between rainfall and the incidence of the disease.

## Method

Based on data obtained from the cases of Leptospirosis contamination - A27, rated by the Manual of the International Classification of Diseases - ICD-10, which were provided by the East Regional Health Surveillance and obtained from the site the DATASUS [11] the state of São Paulo were considered for analysis only data from the E region of São Paulo, between 2007 and 2008, especially the broad area of the Aricanduva river area.

In order to evaluate the most affected region and to associate the epidemiological data of Leptospirosis in the study region, we referred to the physical and rainfall profile of the Aricanduva river, occurrences of flooding in the region and population surveys in the region over the period 2007/2008. This information was collected from the Information System for Management of Water Resources of the State of São Paulo, from the Municipal Planning, from the information systems of the City Hall of São Paulo and from the Brazilian Institute of Geography and Statistics [4,11].

From data of the population survey we considered the indices of the general population and region, not influenced by age or gender, and the study was based on the region of the East zone of São Paulo, specifically in the areas covering the Aricanduva river.

These data were compiled by checking the ratio of reported cases of Leptospirosis with the frequency of rains and floods in the region with the drought. Through information from graphs and tables we prepared results for better visualization and analysis of these indices.

The tables are from general data of Sao Paulo State and Sao Paulo city and we addressed specifically the data of interest to the region of Aricanduva river in the East zone. The overall rates of these references are needed for better epidemiological study of Leptospirosis in the region, as well as the physical properties and rainfall in the Aricanduva river and its surroundings. The values obtained were classified by period of rain and flooding that occurred between 2007 and 2008 and the rate of Leptospirosis infection in the region of Aricanduva river.

We calculated the incidence coefficient. The incidence coefficient expresses the number of new cases of a disease during a defined period in a population at risk of developing the disease. The calculation of the incidence coefficient is the most common way to measure and compare the frequency of diseases in populations. The mathematical expression for the calculation of incidence is:

$$\text{Incidence Coefficient}=\text{number of new cases of a disease in a given place and time in the same place and time of developing the disease and for the same period}\times 10^{n}.$$

Data were obtained from DATASUS, since it is reportable disease to make the diagnosis the health professional must make the notification. Based on these data we made the figures for the eastern district of the city of Sao Paulo, all data. The notification occurs on the place of medical care, i.e. Unity of Basic Health region. Thus, this is related to UBS place of residence of the patient.

## Results

In Sao Paulo city, in 2007, in a population of 10,886,518 inhabitants we observed 29,394 cases of deaths (Table 1). Among these deaths 4,025 were related to infectious or parasitic diseases, which are often related to problems of sanitation or diseases, such as Leptospirosis.

**Table 1:** Information regarding population and mortality in the East zone of Sao Paulo City

**Estimated population in 2007**		
People Residents	10,886,518	Inhabitants
Hospital deaths - infectious and parasitic diseases	4,025	Deaths
Total	29,394	Deaths

The disease incidence decreased between 2007 and 2008 in Sao Paulo State. The incidence coefficient decreased from 1.87 to 1.40 and the incidence of mortality reduced from 14.12 to 12.67.

In 2008, it was reported a population of 41,011,635 inhabitants in Sao Paulo State. Among them it was confirmed 576 cases with 73 deaths recorded. In Sao Paulo city it was found 10.990.2449 inhabitants. Among them it was recorded 172 cases with 33 deaths.

According to Table 2, in 2007, the East zone of São Paulo city presented 205 cases with 42 deaths. In 2008 it was observed 44 cases with 18 deaths.

**Table 2:** **Leptospirose in Sao Paulo and surrounding areas** - **by administrative district and the east zone subprefecture by year 2007**/**2008**

**Adm Distr and Borough**	**CC07**	**IC07**	**D07**	**M07**	**CC08**	**IC08**	**D08**	**M08**	**2007**	**2008**
SP ARICANDUVA	**3**	**1**.**16**	-	-	**1**	**0**.**39**	-	-	**259**,**005**	**258**,**072**
ARICANDUVA	.1	1.06	-	-	-	-	-	-	94,009	93,905
CARRAO	-	-	-	-	1	1.38	-	-	72,997	72,386
VILA FORMOSA	2	2.17	-	-	-	-	-	-	91,999	91,781
SP CID TIRADENTES	**6**	**233**	**1**	**16**.**67**	**1**	**0**.**38**	-	-	**257**,**029**	**265**,**531**
CIDADE TIRADENTES	6	2.33	1	16.67	1	0.38	-	-	257,029	265,531
SP ERMELINO MATAR	**5**	**2**.**42**	**1**	**20**	-	-	-	-	**206**,**470**	**206**,**545**
ERMELINO MATARAZZO	3	2.72	-	-	-	-	-	-	110,419	110,735
PONTE RASA	2	2.08	1	50	-	-	-	-	96,051	95,810
SP GUAIANASES	**5**	**1**.**75**	**1**	**20**	**1**	**0**.**34**	-	-	**286**,**520**	**289**,**874**
GUAIANASES	2	1.89	-	-	1	0.94	-	-	105,684	106,421
LAJEADO	3	1.66	1	33.33	-	-	-	-	180,836	183,453
SP ITAIM PAULISTA	**12**	**3**.**07**	**1**	**8**.**33**	**4**	**1**.**01**	-	-	**391**,**106**	**394**,**513**
VILA CURUÇÁ	5	3.22	-	-	2	1.28	-	-	155,138	156,002
ITAIM PAULISTA	7	2.97	1	14.29	2	0.84	-	-	235,968	238,511
SP ITAQUERA	**13**	**2**.**55**	**5**	**38**.**46**	**2**	**0**.**39**	**1**	**50**	**510**,**101**	**512**,**040**
CIDADE LÍDER	3	2.40	2	66.67	-	-	-	-	124,778	125,589
PARQUE DO CARMO	1	1.48	-	-	1	1.47	-	-	67,634	67,986
ITAQUERA	4	1.90	2	50	1	0.47	1	100	210,956	211,858
JOSÉ BONIFÁCIO	5	4.68	1	20	-	-	-	-	106,733	106,607
VILA LEOPOLDINA	-	-	-	-	-	-	-	-	26,877	26,874
SP MOÓCA	**2**	**0**.**70**	-	-	**5**	**1**.**76**	**2**	**40**	**286**,**503**	**284**,**060**
ÁGUA RASA	1	1.24	-	-	1	1.25	-	-	80,533	79,893
BELÉM	1	2.85	-	-	2	5.78	1	50	35,104	34,610
BRÁS	-	-	-	-	1	4.69	1	100	21,691	21,319
MOÓCA	-	-	-	-	-	-	-	-	58,589	58,046
PARI	-	-	-	-	-	-	-	-	12,356	12,099
TATUAPÉ	-	-	-	-	1	1.28	-	-	78,230	78,093
SP PENHA	**17**	**3**.**58**	**2**	**11**.**76**	-	-	-	-	**475**,**121**	**474**,**920**
CANGAÍBA	6	4.10	1	16.67	-	-	-	-	146,465	147,383
PENHA	6	4.98	-	-	-	-	-	-	120,449	120,013
ARTUR ALVIM	2	1.85	-	-	-	-	-	-	107,979	107,609
VILA MATILDE	3	2.99	1	33.33	-	-	-	-	100,228	99,915
SP SÃO MATEUS	**10**	**2**.**34**	**4**	**40**	**3**	**0**.**69**	**3**	**100**	**427**,**552**	**432**,**949**
IGUATEMI	4	3.13	1	25	3	2.29	3	100	127,796	130,976
SÃO MATEUS	1	0.64	-	-	-	-	-	-	156,696	156,877
SÃO RAFAEL	5	3.50	3	60	-	-	-	-	143,060	145,096
SP SÃO MIGUEL	**13**	**3**.**21**	**1**	**7**.**69**	**2**	**0**.**49**	**1**	**50**	**404**,**923**	**407**,**815**
JARDIM HELENA	8	5.44	-	-	-	-	-	-	147,124	147,923
SÃO MIGUEL	3	3.16	1	33.33	1	1.06	1	100	94,890	94,602
VILA JACUÍ	2	1.23	-	-	1	0.60	-	-	162,909	165,290
SP VL MARIA/GUIL	**5**	**1**.**76**	**2**	**40**	**3**	**1**.**06**	**2**	**66**.**67**	**284**,**337**	**281**,**972**
VILA GUILHERME	2	4.48	-	-	1	2.27	-	-	44,634	44,042
VILA MARIA	2	1.85	1	50	1	0.93	1	100	108,089	107,380
VILA MEDEIROS	1	0.76	1	100	1	0.77	1	100	131,614	130,550
SP VL PRUD/SAPOP	**11**	**2**.**13**	**3**	**27**.**27**	-	-	-	-	**515**,**847**	**514**,**622**
SAPOPEMBA	9	3.11	3	33.33	-	-	-	-	289,069	289,599
SÃO LUCAS	1	0.76	-	-	-	-	-	-	131,520	130,573
VILA PRUDENTE	1	1.05	-	-	-	-	-	-	95,258	94,450
**Total**	**205**	**2**.**38**	**42**	**20**.**49**	**44**	**0**.**51**	**18**	**40**.**91**	**8**,**609**,**028**	**8**,**645**,**826**

Confirmed Cases (CC, per 100,000 inhabitants), Incidence Coefficients (IC, %), Death (D) and Mortality (M). Data source from DATASUS [11].

It was observed that between 2007 and 2008, there was also a significant decrease in Leptospirosis cases in the East zone of Sao Paulo. The incidence coefficient decreased from 2.38 in 2007 to 0.51 in 2008. However, mortality coefficient increased from 20.49 in 2007 to 40.91 in 2008.

Table 3 indicates that among total cases reported in the East zone of Sao Paulo the area of Aricanduva river and surroundings areas presented 90 cases with 22 deaths in 2007 and 22 cases with 12 deaths in 2008.

**Table 3:** **Leptospirose in Sao Paulo and surrounding areas in 2007**/**2008**

**Adm Distr and Borough**	**CC07**	**IC07**	**D07**	**M07**	**CC08**	**IC08**	**D08**	**M08**	**2007**	**2008**
**SP ARICANDUVA**	**3**	**1**.**16**	-	-	**1**	**0**.**39**	-	-	**259**.**005**	**258**.**072**
**SP ITAQUERA**	**13**	**2**.**55**	**5**	**38**.**46**	**2**	**0**.**39**	**1**	**50**	**510**,**101**	**512**,**040**
**SP MOÓCA**	**2**	**0**.**70**	-	-	**5**	**1**.**76**	**2**	**40**	**286**,**503**	**284**,**060**
**SP PENHA**	**17**	**3**.**58**	**2**	**11**.**76**	-	-	-	-	**475**,**121**	**474**,**920**
**SP SÃO MATEUS**	**10**	**2**.**34**	**4**	**40**	**3**	**0**.**69**	**3**	**100**	**427**,**552**	**432**,**949**
**Total**	**90**	**2**.**30**	**22**	**24**.**44**	**22**	**0**.**56**	**12**	**54**.**55**	**3**,**916**,**564**	**3**,**924**,**082**

Data source from DATASUS [11].

In the region spanning the Aricanduva river we also observed a decrease in cases of Leptospirosis. It was reported an incidence coefficient reduction from 2.30 in 2007 to 0.56 in 2008 and a increased mortality from 24.44 in 2007 to 54.55 in 2008.

Tables 4 and 5, mentioned the districts of Sao Paulo East zone neighborhood with spots of flooding identified by the DATASUS [11].

**Table 4:** Records of flooding areas in 2007 and 2008 in Sao Paulo East Zone and Sao Paulo City

**2007**	**JAN**	**FEB**	**MAR**	**APR**	**MAY**	**JUN**	**JUL**	**AUG**	**SEP**	**OCT**	**NOV**	**DEC**	**Total**
	**12**	**23**	**22**	**25**	-	-	**1**	-	-	**4**	**4**	**11**	**101**
**2008**	**JAN**	**FEB**	**MAR**	**APR**	**MAY**	**JUN**	**JUL**	**AUG**	**SEP**	**OCT**	**NOV**	**DEC**	**Total**
	**19**	**50**	**7**	-	-	-	-	**3**	-	**2**	**8**	**9**	**98**

Data were obtained from the Database of the Unified Health System (DATASUS) [11].

**Table 5:** Leptospirose cases reported in Aricanduva river area and Sao Paulo East Zone in 2007 and 2008

**2007**	**JAN**	**FEB**	**MAR**	**APR**	**MAY**	**JUN**	**JUL**	**AUG**	**SEP**	**OCT**	**NOV**	**DEC**	**2007**
	5	8	14	7	11	13	2	2	2	1	5	3	73
**2008**	**JAN**	**FEB**	**MAR**	**APR**	**MAY**	**JUN**	**JUL**	**AUG**	**SEP**	**OCT**	**NOV**	**DEC**	**2008**
	0	3	1	4	2	5	2	1	2	1	1	1	23

**Figure 1:**
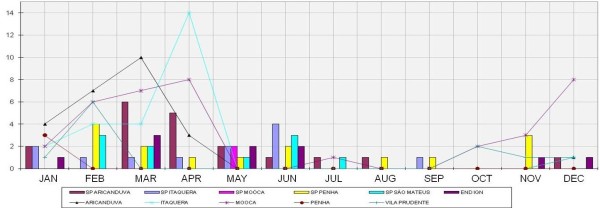
**Flooding points and Leptospirosis cases**, **2007. **Data source from DATASUS [11].

**Figure 2:**
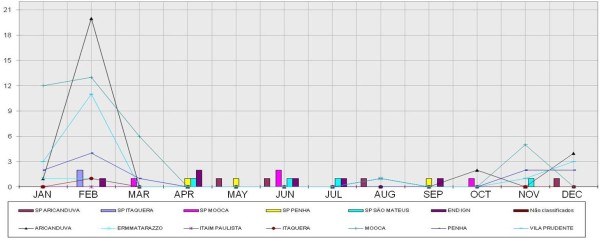
**Flooding points and Leptospirosis cases, 2008. **Data source from DATASUS [11].

Figure 1 and Figure 2 present the columns of confirmed cases of Leptospirosis. Data were obtained from the Database of the Unified Health System (DATASUS) [12]. The figures are represented by lines of indices of flooding areas. These data were collected from the site of the Center for Emergency Management of São Paulo (CGE) [13].

## Discussion

This study was undertaken to investigate epidemiological data of Leptospirosis cases in the East zone of Sao Paulo city, SP, Brazil and also to evaluate the association between rainfall and the incidence of the Leptospirosis. The comparative study of 2007 data, among the cases of Leptospirosis and the flooding that occurred in the region of Aricanduva East Zone and around areas has shown a direct and important water contamination. Records of flooding points and dates of the reported cases in the region showed a direct relationship from which the period of higher rainfall was coincident with an increase in cases. The annual record of the municipality and the region under study and rainfall regions also allowed an association between the values. There was a substantial decrease between 2007 and 2008 in the records of cases of Leptospirosis in the region Aricanduva and the East Zone, however, an increased rate of mortality of the disease was observed.

In our study, the notification of Leptospirosis cases was compromised because the incubation of the bacteria may be long and the patient often seek health care from the onset of symptoms. Leptospirosis may be easily confused with other diseases and its diagnoses require laboratory monitoring of classical clinical and epidemiological data. Often, the confirmation of Leptospirosis appears only one time after contact with the contamination.

The data obtained in spite of confirming a direct contamination by water, it may not determine the actual rate of cases on the outskirts of the contaminated region of the Aricanduva river. Since these data are variable because many of the itinerant confirmed cases of hospitalization obtained in DATASUS sites [11] in 2009 are admission and residence, which does not specify the exact site of infection, not all patients seeking health services occurred near the site of exposure. Moreover, not all cases were reported, many cases are unnoticed and may be confused with a bad flu and laboratory tests can not always detect the classical bacilli *Leptospira*. The time for diagnosis of the disease is very important and laboratory techniques currently developed assist in achieving a more rapid, accurate and makes the treatment more effective [13-16].

Previous studies have already investigated this issue [17-19]. In the study of de Oliveira et al. [20], data on the morbidity and mortality of leptospirosis was collected from Rio de Janeiro’s Municipal Health and Civil Defense Department. The authors concluded that there is a direct correlation between the incidence of leptospirosis and rainfall. Nevertheless, they emphasized that the oscillation of the number of cases is not only determined by rainfall, since other factors influence this dynamic, such as sanitation, in addition to environmental and social factors. Another study [21] indicated that for every 20 mm precipitation, there was an average increase of 31.5% in hospital admissions in Sao Paulo. A different group of researchers [22] reported in Sao Paulo that in the rainy season, it also increases in other districts, probably due to the proximity of rivers and streams while in the dry season, the localities where cases appear coincide with the areas of poorest housing conditions.

As a main finding, our epidemiological study indicates that the Aricanduva river region and surrounding areas are worth to be further investigated. We also suggest additional projects in order to attenuate the flooding caused by rainfall in this area and, as a consequence, reduce the cases of Leptospirosis and others diseases caused by similar mechanism.

## Conclusion

There was a substantial decrease of Leptospirosis cases in 2008 compared to 2007 in the region Aricanduva, however, there was an increased rate of mortality caused by Leptospirosis. The associations between the indices of flooding and confirmed cases showed that preventive measures are necessary in order to avoid exposing the community to waterborne diseases such as Leptospirosis and thereby prevent possible outbreaks of disease.

## Competing interest

We declare no conflict of interest.

## Authors’ contribution

KEM, ALAF, LZC, KCR, LAA, VBCJ, ECP, LCC, DF, RC, LCdA, VEV, SNBL, FCG and FLAF participated in the acquisition of data and revision of the manuscript. KEM, LCdA, VEV and FLAF determined the design, interpreted the data and drafted the manuscript. All authors read and gave final approval for the version submitted for publication.
